# Making space to learn about teaching: expanding teaching horizons through postgraduate education

**DOI:** 10.1007/s10459-022-10144-4

**Published:** 2022-08-09

**Authors:** Gillian Aitken, Tim Fawns, Katey Warran, Derek Jones

**Affiliations:** Edinburgh Medical School: Medical Education, University of Edinburgh, Chancellor’s Building, EH16 4SB Edinburgh, Scotland

**Keywords:** Conceptions of teaching, Teaching identity, Postgraduate education, Faculty development, Complexity

## Abstract

Clinicians develop as teachers via many activities, from on-the-job training to formal academic programmes. Yet, understanding how clinicians develop the sensibilities of an educator and an appreciation of the complexity of educational environments is challenging. Studies of teacher development have maintained a relatively narrow definition of educational practice. A more expansive view encompasses clinical teachers’ roles in relation to elements beyond learners or content, such as the cultures and other structures of healthcare institutions. In our online Postgraduate Certificate in Clinical Education, space and structure are intentionally created for teachers to think and talk about education with colleagues in other disciplinary contexts. We interviewed 17 students about how their approaches to teaching had changed over a year of part-time study, using their teaching philosophies, written at the start of the programme, as points of contrast. We took an abductive approach to data analysis, drawing on the literature and, unavoidably, our own reflexive interpretations of our practice outside of the research context, such as conversations with students and colleagues; our experiences of teaching and our concurrent research and scholarship. Our themes of repertoire building, perspective shifting, embodied practice, and appreciation of context, describe the increasing complexity of individuals’ considerations of teaching. We use our analysis as the basis for a discussion of the blurring of boundaries between staff and students on such programmes as both groups are engaged in an ongoing continuum of development as all teachers, continue to be learners of educational practice. These insights can inform the ways in which postgraduate programmes can make space for clinical teachers to share and reflect on practices, perspectives and contexts.

## Introduction

We are rarely asked how we think about teaching, or how we learn to teach, but our conceptions of teaching affect how we teach and influence our students’ learning (Martin et al. 2000, Trigwell & Prosser 1996, Entwistle et al. 2000). Conceptions of teaching are often idiosyncratic and poorly articulated (Young, 2008), context dependent (Stenfors-Hayes et al. 2011), resistant to change (Entwistle & Peterson 2004), or in a constant state of messy evolution (Squires 1999). Previous work has attempted to categorise conceptions of teaching as primarily teacher-centred (transmission of knowledge) or student-centred (facilitation of learning) (Postareff et al., 2007), or as learning-focused or content-focused (Postareff & Lindblom-Ylänne, 2008). In a healthcare context, Keskitalo et al. (2013) investigated clinical educators’ perceptions of teaching, reporting three discrete categories: communicating knowledge and skills; developing skills and understanding; and facilitating learning. While these categories are useful to those starting to think about their teaching, the focus is on a narrow view of what the teacher does, and of what learning is. What is missing is a more expansive sense of the possibilities of generating and applying knowledge, and of the teacher’s position in relation to elements other than learners or content, such as the cultures and other structures of educational or disciplinary institutions that are so influential in clinical teaching.

The systems in which teaching occurs shape how teaching happens, and how it is understood. The time constraints faced by clinical teachers, for example, may tempt those delivering educational programmes to focus on the acquisition of teaching techniques, such as tips for small group teaching or delivering a lecture. Whilst these skills are undeniably useful, they fail to take into account that teachers and teaching are situated within complex settings and wider, social and political contexts, where one-size will rarely fit all (Fawns et al., 2021a; Fawns & Sinclair, 2021). It is these wider influences that create the pressures on teaching that lead to its instrumentalization (i.e. its separation from professional practice or its division into reductive components and methods) (e.g. Naidoo & Williams 2015; Ransome, 2011). Moving beyond instrumental views of teaching and teacher develop requires developing a sensibility around how teaching and learning are shaped by context, student-teacher relationships, and activities other than formal teacher-student interactions.

This paper features a reflexive exploration of how an online Postgraduate Certificate in Clinical Education can make space for the development of more expansive conceptions of learning and teaching in clinicians. While clinicians who elect to undertake a formal programme of study in clinical education might be regarded as atypical, with a keen interest in the topic, there is no doubt that numbers undertaking such study are increasing (Tekien and Harris 2012). Online programmes, in particular, afford clinicians the opportunity to combine study with clinical duties (Aitken 2020). Such programmes have been conceptualised as spanning boundaries, situated as they are in the complex and overlapping areas of healthcare education and practice (Aitken & O’Carroll, 2019). There is no formal curriculum or regulatory body which oversees the content of such programmes which is largely led by market forces and the expertise of programme staff, who often have a clinical background (Aitken & O’Carroll, 2019) and—like their students—are, themselves, clinical educators.

## Methods

### Setting

The fully-online Postgraduate Certificate in Clinical Education at the University of Edinburgh allows clinical educators from a range of disciplines (e.g., medicine, veterinary medicine, nursing, dentistry, allied health professions) and an international range of locations, to study together. Our programme emphasises community building, flexibility, critical appraisal, and sharing and applying theoretical ideas to the students’ diverse professional disciplines, experiences and settings (Aitken et al., 2019; Fawns et al., 2021b).

At the time of writing, there are over 150 first year students on our programme, most of whom study part-time, combining study with clinical work. The first-year consists of three individual courses, all 10 weeks in length, that involve a mixture of real-time videoconference conversations, discussion boards, learning activities and supporting learning materials. At the time of the study GA was the lead for course one (Principles of Teaching and Learning), TF the lead for course 2, (Assessment, Standard Setting and Examinations) and DJ the lead for course 3, (The Curriculum). GA and DJ have considerable clinical expertise as a dietitian and occupational therapist respectively, TF has a professional background as a learning technologist. The tutors have been involved with this programme for a combined total of 23 years and have a variety of teaching and learning expertise gained over many years in diverse settings. They were supported in data collection and analysis by a PhD intern (KW) who was funded by an internal teaching grant.

We oriented our research around the following broad research question:

How does postgraduate study help clinical educators learn about teaching?

We approached this question by looking across the year of part-time study, using our participant’s early teaching philosophies, written at the start of the programme, as a point of contrast with their later understandings of teaching and learning.

### Data collection

The data for the present study comprise two sources:

1) For each participant, a formative “philosophy of teaching” assignment, completed in week 5 (one student, Frank, had not completed this work due to recognition of prior learning). This involved a short articulation of what was important to them in relation to teaching and learning. Tutors provided verbal and written feedback around the thoughts and questions these assignments raised for them. Tutors also shared a written programme philosophy with the students, the production of which was instrumental in our motivations to undertake this study.

2) A follow-up research interview, at the end of the first year, when students could either graduate with a Postgraduate Certificate in Clinical Education, or continue on to complete a Postgraduate Diploma. During each interview, the participant’s earlier philosophy of teaching was used as a prompt to discuss how their thinking had changed over the year.

There was approximately nine-months between assignment submission and interview. Interviews lasted between 30 and 40 min, each conducted via videoconferencing software (Adobe Connect) by a member of the research team (GA, TF, KW or DJ). Interviews were transcribed verbatim by an external service. An interview protocol, including details of the questions asked, is provided as supplementary information.

### Data analysis

We employed a thematic analysis approach, following Braun & Clarke (2006), by generating initial codes relating to ways that the participants’ understandings of teaching had changed. Our approach proceeded abductively, with regular conversations amongst the researchers, questioning our interpretations of the data, and tentatively testing out a range of theories and frameworks from the literature (see the Discussion for those deemed most relevant to the final results). Initial analysis was then carried out using NVivo 12 by KW, during which codes were discussed and refined in regular meetings with the whole team (GA, DJ, TF, KW). Our analytical process included several stages of conceptual mapping, in consultation with the team, to explore connections between themes and theoretical ideas. We stayed close to the data in the initial coding of each transcript and then, in the latter phase of analysis, our approach was to interpret across the data, rather than to separately analyse excerpts. In places, this means we have included fewer quotations in order to make space for more justification of our interpretations.

In abduction, an interpretation cannot be definitively correct or right; it is valid by being useful (Reichertz, 2004). The final set of results is always one of a number of possible cases, chosen on the basis of how it fits the data (Veen, 2021). As Veen notes (p1181), abduction involves “taking creative leaps from empirical observations, with the acknowledgement that there are always other possible theories and explanations”. Abduction gives us a way of explaining unexpected data by elaborating, adapting, and combining existing concepts. Therefore, the knowledge and preconceptions of researchers are an inevitable aspect of abductive inference (Richardson & Kramer, 2006). Indeed, this is key to constructing explanations that are attuned in relation to empirical data. Coffey & Atkinson (1996 p. 157) argue, “we use concepts, theories and ideas constructively and creatively… Regularities in data – whether of form or content – must be associated with ideas that go beyond those data themselves.” Thus, discovery and justification go hand-in-hand (Reilly, 2019; Timmermans & Tavory, 2012), and dialogue amongst the team, as well as conversations with students outside of the research process, was a significant part of this.

### Reflexive considerations

During the analysis, we considered the ways in which our participants’ accounts indicated a progressive appreciation of the structures, environments, and cultures in which their teaching practice took place. As such, our analysis focuses on how our participants understood the practices and values of teaching, and their entanglements in the complex contexts of professional learning. In this analysis, we cannot separate out ourselves as researchers and as teachers who concurrently research, practice, and engage in dialogue with students and colleagues about the issues we are researching. As teachers, we are, ourselves, always learning through these different and overlapping avenues of practice, dialogue and research. Our approach to our learning and teaching is sociocultural (Vygotsky, 1978) embracing the value of social interaction in the development of clinical educators.

Our students come with a diverse range of skills and experience. Many are at the start of their clinical and teaching careers, while others are very experienced in one or both aspects. The programme is also taught by a multidisciplinary group who share many of the concerns of our students. Indeed, our motivation for this study came from the ongoing discussion and questioning amongst the programme team about our own development as clinical educators and how this influences the design and running of the programme. We are curious, not only about how our students learn about their place in the clinical education ecosystem, but also about how we can learn about our own. This context requires a reflexive approach to data generation and analysis, as well as a rethinking of the boundary between teacher and learner, and researcher and participant, as we consider what can be learned from teachers talking to their students, who are also teachers, about what it is to teach and to be a teacher.

Thus, our thematic framework is unavoidably shaped by the intersubjectivity of research interviews (Mishler 1991) between participants and interviewers who are mutually engaged outside of the research context in learning about education. It was inevitable that our own positions influenced the conversations within the interviews and our analysis of the data afterwards. There is much of ourselves in how our students see education, in our conversations with our students in the interviews, and in the ways in which we have interpreted and organised the data. Yet, by positioning ourselves as, to some extent, participant-researchers who reflect on our own practice at the same time as reflecting on that of our student-colleagues (Fox et al., 2021), we also gain an opportunity to consider, from an insider perspective, the entanglements and erosion of boundaries between what are usually considered distinct categories: teacher / student, and researcher / participant. The implications for interpretation are that the particular insights presented in this paper may be contingent on the combination of our approach to teaching these participants, and our role as educator-researchers more broadly. Our results should therefore be read, not so much as a generalisable account of how conceptions of teaching expand through postgraduate education but as one case of a wider set of possible expansive conceptions of teaching and how this form of postgraduate education created space for those to arise.

### Ethical considerations

#### Ethical approval

was acquired from the local School of Education Research Ethics Board. Taking part was voluntary, participants gave informed consent, and data were de-identified and anonymised. Students had completed their studies (and assessments) before the interviews were undertaken.

## Results

Seventeen students agreed to participate in this research (11 in academic year 2017/18, and 6 in academic year 2018/19). Information about the backgrounds of the study participants is provided in Table 1.

**Table 1:** Participant Information

Pseudonym	Professional background	Location	Age	Gender	Years of professional practice
Alice	Paediatrician	Southern Africa	30–39	F	10–15
Carol	Emergency physician	UK	20–29	F	0–5
Elaine	Disabilities nurse	UK	30–39	F	10–15
Giselle	Clinical teaching fellow	UK	30–39	F	10–15
Kathy	Advanced nurse practitioner	UK	40–49	F	25–30
Ruth	Education manager	UK	40–49	F	10–15
John	Physician	East Asia	30–39	M	5–10
Mary	Paediatrician	Europe	40–49	F	15–20
Betoul	Nurse	Southern Africa	50–59	F	25–30
Farah	Pharmacist	Southern Asia	40–49	F	15–20
Scott	Dental surgeon	UK	40–49	M	15–20
Mohamed	Medical trainee	UK	30–39	M	0–5
Ayesha	Clinical Instructor	UK	40–49	F	5–10
Frank	Physician	UK	60–69	M	10–15
Sanjit	General surgeon	Southern Asia	40–49	M	15–20
Lisa	Oncologist	UK	50–59	F	30–35
Jess	Obstetrician	UK	40–49	F	10–15

There was a slightly higher proportion of female (12) and medically-qualified (11) participants than we might expect in a typical student cohort. The academic performance of both cohorts represented here is typical.

Our participants’ developing understanding of teaching included not only particular teaching practices and theories, but also developing sensibilities and embodied forms of knowing. Across the two data sources, there was an overall shift in language between the formative teaching philosophy assignment (TP) and the research interview (RI), with the language in the former generally couched in aspirational terms (as areas to develop rather than qualities already possessed). There was also evidence of an overall shift in the participants’ holistic perspectives on education (beyond temporary flirtations with various theories) that opened up meaningful new possibilities for observation, widening the scope of what was available for reflection. This is also likely to be related to students increasing confidence with educational terminology.

Four themes are presented here, according to increasing levels of complexity. Not all themes were evident for every student, and most teaching philosophies focused on the first and second themes. In the interviews, we had greater opportunity to explore the ways in which participants were expanding their perspectives, embedding educational principles throughout their practice (theme 3), and coming to appreciate the ways in which their capacity to do that is contingent on how they can function within complex systems (theme 4).

Each of these themes is explored in more detail below.

### Repertoire building

An important aspect of our participants’ development as teachers was the building-up of a repertoire of skills, methods, theories, and growing confidence with the language of education. The potential to draw on different teaching methods, for example, was seen by some as part of one’s credibility as a teacher. Exposure to, and consideration of, different methods of teaching—through readings, discussions with peers and modelling by programme tutors—allowed participants to think about and try new approaches and, thus, engage in a wider range of experiences. Those experiences could also be conceptualised through the use of different and, potentially, multiple theories learned during their postgraduate studies. As such, the building of a repertoire of theories was an important component of an expanding methodological range. Scott shows how an expanded repertoire could support the confidence to be versatile.

*I was looking more for, am I doing right? Is there evidence for me doing it in a certain way? Now I look at it and I go, actually I can now cherry-pick the aspects that I want from different approaches… I am now looking at it and trying to adapt what I can do. (Scott, TP)*

Skills and methods were not just ends in themselves but also important building blocks for more sophisticated forms of knowledge and expertise. Through their end-of-course assessments, participants were required to articulate theories and methods as they related to their practice contexts. This helped them to consider the material requirements, constraints and implications of their favoured methods, and to critically appraise the application of theory to context. For example, Jess spoke in her interview of the physical and cultural barriers to setting up an interprofessional teaching session for haematologists and obstetricians.

*Both teams are really important to managing pregnancies…. The barriers are trying to bring two different specialties together… they are very opinionated about how they manage patients so there will be barriers there. The other is time and finances. How do we get them to sit together, what time would be suitable for them? Would we have physical availability of classrooms, technology… there are a lot of barriers here. (Jess, RI)*

This example shows how developing teaching methods is more than knowledge acquisition, and that the resources that teachers employ are more than tools or objects for manipulation. Logistics and theory are interwoven, and there is an expansion of possibilities but also a convergence of integrated ideas as theory is narrowed by practice. Thus, part of repertoire building is an appreciation of how methods must be reconciled with the material and cultural environments in which they are executed and captures the complexity and potential sensitivities of teaching in such environments.

### Perspective shifting

Many of the teaching philosophies, written at the beginning of the programme, contained narrow and linear conceptions of education. For example, in her philosophy, Giselle saw teaching as a means of “optimising learning” but without offering a clear sense of what this meant in reality, or questioning if such a thing is actually possible. There was often a simplistic conception of interacting elements: students, teachers, and the learning environment. Learning environments were generally discussed as something provided by teachers to students, not recognising that teachers and students are part of the environment and that they create it together. In most philosophies, even those that advocated for student-centred education, the focus was firmly on what the teacher was doing, with much discussion of the multifaceted role of the clinical educator and the important role modelling provided by clinical educators.

Between the written philosophies and interviews, there was an apparent, overall expansion of the possibilities of learning and, therefore, also of teaching. In their interviews, some participants spoke of knowledge, teaching, and the role of the teacher as less bounded. For example, Ayesha had come to see learning and teaching as ongoing accomplishments: one never finishes learning, and one never finishes becoming a teacher.

*The thing about learning and teaching, it’s like everything else, you’re never finished. There’s always something new… there’s always things you can learn, either from the way that your students have responded or the way they’ve challenged you… it’s a never-ending journey, actually. (Ayesha, RI)*

For Frank, there was a mutuality between teaching and learning, where teachers needed to understand the point of view of the learner, but learners also benefited from understanding the approach taken by the teacher. He spoke, for example, of active learning methods only working if the approach was understood by both teacher and learner.

*This concept of active learning is something that needs to be learned, not only by the educators but also by the learners themselves. (Frank, RI)*

His plans to help students develop understandings of how to engage with his teaching came from a shift from seeing a teacher as one who transmits information to someone who has a role in helping prepare students for future challenges of complex practice.

*For example, clinical ward rounds when we involve the students in the clinical environment, I realised that everything is about the learning process. (Frank RI)*

Beyond theoretical knowledge, participants learned through shifting their perspectives on what teaching is, how learners learn, and what is important within education. For many participants, this entailed a recognition that teaching is less rigidly defined by particular methods or approaches, or restricted to particular, discrete teaching sessions, or confined to particular spaces and times.

*If I were to rewrite the philosophy, I’d be more mindful of how liquid and how fluid learning and teaching can be, and how there are many historical ideas which kind of form the foundation, but still have variation depending upon the learners and the teachers and the environment. (John, RI)*

For some, teaching had come to be seen as shaped by the context, purpose, and the activities of the teachers and learners, not just within formally-scheduled sessions but whenever teachers engaged with students. Lisa also conveyed this notion of learner and teacher learning and practising together, referencing an “apprenticeship model” and blurring of boundaries between the traditional conceptions of teacher and student.

*As a senior clinician, teaching is about enabling trainee doctors and undergraduate students to develop the knowledge, skills and attitudes required for them to succeed in their careers by positively and enthusiastically sharing my knowledge and learning from my clinical experience. If as a learner and teacher, you can describe the “why”, it makes it so much easier to engage with the topic. However, learners often teach me as much as I learn. (Lisa, TP)*

All participants discussed how their ideas about teaching and learning had expanded as a result of their studies, often in ways they could not have predicted at enrolment. Their studies had encouraged them to widen their gaze and better appreciate the complexity of the teaching they were involved in, rather than focusing on their own formal educational activities. This relates not just to the content of the teaching and its delivery but the wider social, material and environmental factors that influence all clinical teaching.

### Embodied practice

Some participants found that, having learned more about methods and theories, and having developed a more complex perspective on education, that there were now “more things to think” (Farah) than there used to be, or that there had been a shift from enacting routinised approaches to “constantly thinking about what I am doing” (Mary). This expansion of thinking about educational practice and theory suggests a developing, embodied capacity for reflection and adaptation. However, language often remained aspirational, as participants recognised the need to go beyond traditional teaching and assessments but struggled to do this consistently. There were some important barriers to embodying theoretical knowledge.

Firstly, it was possible to talk of progressive approaches to teaching in one sentence and to convey a different reality in another. For example, the dominant, espoused narrative of student-centred education was at odds with the descriptions of practice of several of our participants in which tasks, groupings, timings and resources were chosen for the students. It was also possible to understand some of the complexity of teaching, or of knowledge, but not yet be able to apply that understanding to practice. For example, Scott recognised the need to change his teaching in relation to the specific challenges of his students, but did not yet see past the method he had been using (Powerpoint presentation) to consider alternative approaches.

*It’s not doing a PowerPoint presentation and keeping it on your hard drive and every year rolling it out and giving them the same presentation. It’s looking back and going actually those people didn’t get it and this was the reason why. So, what can I do in my presentation that’s going to change it? (Scott, RI)*

Alice gave an example of being in a liminal space between knowing something (that knowledge is not fixed) and being able to act on it as a teacher, learner or practitioner.

*It’s something that I’m continually trying to figure out how to get past, just the concept that knowledge is finite… that’s not the case. Knowledge changes… every [patient] is different. (Alice, RI)*

Through reading and discussing research literature, and exposure to the alternative approaches of tutors and a diverse set of peers, studying on their postgraduate programme created a means of escape, for some participants, at least, from the cultural loops in which teachers can become trapped. For instance, in contrast to some of the more fixed notions of learning environments conveyed within the earlier teaching philosophies, Lisa was now able to appreciate

*… people’s different cultural backgrounds… I didn’t take as seriously before. But now that I’m reading up on the literature, I’m understanding why it is that I need to be more aware of it… how a student might interact with their colleagues or the teacher or the environment, you have to take all of these things into consideration. (Lisa, RI)*.

Some participants conveyed an awareness of how to engage with educational challenges holistically, with the situation at hand not just an additional consideration but an integrated part of their pedagogical approach. Giselle gave an example of a process of attunement rather than simply tweaking a pre-defined method.

*Think about how I was doing it, why I was doing it, what they needed and what I wanted and, you know, sort of how you align those things and start to shape it towards it, as opposed to just, yes, fitting into a role that was given to you a bit more. (Giselle, RI)*

This holistic sensibility, where different elements of a teaching repertoire are integrated, allowed participants to talk of aligning their practice with an expanding perspective on what is involved in teaching and the confidence to flexibly adapt their teaching to best fit the needs of any given context.

### Appreciation of context

This theme concerns an expanding appreciation of the wider contextual influences on teaching practice. Where the previous theme concerns change in embodied ways of knowing, this theme concerns changes in reflexive awareness of the teacher’s situation within institutions and cultures. The initial teaching philosophies focused more on interactions between teachers and students, the research interviews allowed more space for discussions of culture, politics, infrastructure, and economics. To some extent, these more nuanced discussions are likely to be to do with the format, but participants also talked of their appreciation of context developing during their studies. John spoke of a newfound appreciation of diversity

*and the idea that one size doesn’t fit all and that what may work in one institution with one group of students and in one system may not work as well in another setting. (John, RI)*

Ruth talked of a shift from a previous unawareness, before starting the programme, of political and cultural influences on teaching.

*It didn’t occur to me at all. I at no point thought, oh you know, these complex hierarchies are making it difficult for me to feel comfortable therefore I’m falling back on the way I used to feel when I was a kid… I didn’t realise… I had no concept of how the environment, how the social structure, how my past experiences of education, or how any of that was affecting me... (Ruth, RI)*

In many cases, engagement with context reflected a recognition of obstacles and barriers to change. Betoul was aware of the rationale for a student-centred approach but struggled to put this into practice because of institutional culture and limited resources.

*That’s the tricky part... It has to include not only the faculty or the teacher but the institution as well. If the institution fosters a culture where students have the resources and all of these different things in order to self-direct learning, then the faculty can provide the support and guidance that is needed. However, if that culture, which will include resources and support and all of these things, is non-existent, then it’s kind of hard for you to just say to the student, “well these are your materials, go, learn, and I’ll be there to guide you (Lisa, RI Participant 16).*

While not every participant explicitly demonstrated an awareness of contextual issues within their philosophies or interviews, we believe that this theme is relevant to all participants as no teaching occurs in a vacuum. Some appreciation of context was still evident in the ways participants talked about teaching in relation to competing priorities such as patient care, workload or physical spaces.

This developing understanding was not always comfortable. Some participants felt an increasing sense of isolation as they were unable to bring others on board with their perspectives, values, and goals. Tension was apparent between what participants now saw as important within teaching, and what they felt able to apply and embody in their teaching practice.

*…trying to work out how to stop doing what I now believe is not the best approach… how to put that into practice in an area where I’m the only person who was possibly thinking like that (Kathy, RI)*

It was evident, therefore, that this was the most complex of our four themes, because acting on an expanding contextual understanding required not just individual development, but cultural change. However, some participants demonstrated how understanding their constrained position within the cultures and structures of an institution could help them to realise some of the less direct activities that could facilitate change over time.

*We have actually created a group of medical educators and we have convinced the administrator to form a unique medical education unit that continuously looks into the curriculum, the teaching and learning process, the assessment methods and evaluate and do some form of improvement…, if they don’t increase the resources and facilities, it’s going to be challenging (Sanjit, RI)*

Sanjit’s collective activity went beyond methods, to making changes to the local teaching cultures and structures. Different positions within an institution shaped what it was possible to do and also the vantage point from which the educational context could be understood. Seniority, which varied across participants, influenced the extent to which they were in a position to enact change. Institutions were described as “hierarchical” and “resistant” to change in relation to teaching, and logistical challenges included were lack of resources, time and finances. These challenges were often connected to the lack of value given to education within the culture of the organisation. In one of the more forceful expressions of this, Ruth suggested:

*It would be nice to work in an environment where you don’t have to feel as though your role has no value (Ruth, RI)*

Whatever position a participant occupied within the local organisation, beliefs could not be embodied in practice without institutional support, which, in turn, might require forms of work that were removed from what is normally considered “teaching”, and which involved influencing others to contribute to reform.

## Discussion

Our themes represent the expansion of participants’ understandings of *what teaching is* over the first year of their online postgraduate studies. In organising our interpretations of the data in this way, we have departed from categorisations of teaching in terms of skills and attributes. The initial theme—building a repertoire of methods and theories—is representative of common approaches to faculty development, where there is emphasis on the technical and content aspects of teaching. As Shulman (1986, 1987) noted, decades ago, to stop there is to take a narrow perspective on teaching and teacher development. Shulman (1987) argued for seeing subject matter, knowledge of teaching methods, and pedagogical reasoning, as integrated into what he called “pedagogical content knowledge”. Our analysis breaks down this integration of knowledge into four themes of increasing complexity.

Teacher development, it seems, includes the confidence and capacity to take alternative perspectives, to embody values and perspectives within situated practice, to build awareness of complex interrelations, and to integrate into and shape complex systems. Our themes help us to consider development also in terms of how teachers think about teaching, without discounting the need to build up knowledge of methods and theories. Indeed, repertoire building may be necessary to allow comparison between approaches and, thus, enable the changing of perceptions (and the possibilities for what can be thought about), seen in our second theme. The third theme, embodied practice, goes beyond what is explicitly thought about within teaching, to how a more holistic educational sensibility is enacted by diverging from specific methods and concepts, and attuning to situated conditions. The final theme—appreciation of context—explains participants’ consideration of the structures, systems and cultures in which their practice was located, and of their capacity to adapt to, and shape, those elements. It recognises the considerable constraints within which teaching is situated, and the impossibility of direct control over many important aspects of educational practice.

Awareness of complexity, in many ways, is the basis for meaningful action, as well as a potential catalyst for changing one’s thinking. For example, considerations of inclusivity and diversity might come more clearly into view as one’s perspective shifts, but it is only with increasing awareness of the subtleties of contextual, cultural, political and social factors that these considerations can begin to weave themselves throughout one’s practice and discourse. The creation of space within postgraduate programmes for dialogue around multifaceted challenges, reflexive interrogation of teaching and workplace settings, and comparison across settings is conducive to the development of an appreciation of complexity (Rupert, 2018). Yet, we acknowledge that the realisation of theme 4 may be beyond what can be taught and learned through postgraduate educational programmes, because it relies on students working out their own ways to effect change as integrated parts of their workplace environments, and will need to be done over a longer time than this period of study. Theme 4, in particular, represents a direction of travel without a final destination.

The parallels between teachers and their learners become clear in reviewing our themes, as our participants shift away from teaching as methodical instruction to teaching as an embodied way of being that is significantly comprised of learning and development. For example, there seemed to us to be a parallel between the shifting epistemologies of some participants (e.g., toward non-linear, non-finite knowledge) which came about during our programme, and their developing understandings of teaching as “fluid”, collective, ill-defined, and as intimately intertwined with learning (of both students and teachers). This notion echoes Lave’s (1994) discussion of the value of challenging the distinction of teachers from learners within a community of practice: “the social-cultural categories that divide teachers from learners in schools mystify the crucial ways in which learning is fundamental to all participation and all participants in social practice” (p. 157). This dichotomy also presents a divide between formal and informal learning practices, one which comes under scrutiny in relation to our participant’s expanding understandings of the pervasive influence of background structures, policies, cultures, technologies and environments. Indeed, focusing on how educators are positioned in complex contexts can help to broaden out conceptions of teaching to beyond the individual. This is likely to include certain forms of political activity, such as the questioning of power structures and “conditions of marginalization” (Conrad & Openo, 2018), primarily represented within our data in terms of an awareness of and, sometimes, resistance to, the constraints of hierarchies and cultures.

### Implications and recommendations

This work has implications both for those who run postgraduate programmes and those who choose to study them. It also offers the wider community of healthcare educators an insight into such programmes.

By moving beyond the categorisation of conceptions of teaching and considering how educators develop (in this case, in relation to a programme of postgraduate study) the themes we have identified could usefully inform the planning and implementation of faculty development programmes for clinicians who teach. For example, our results remind us, as curriculum developers, of the importance of giving students access to diverse examples of practice, along with a critical, theoretical consideration of these. Applying multiple lenses can highlight otherwise invisible issues and overcome some of the limitations of each perspective (Hager and Hodkinson 2009). Thus, we see value in encouraging educators not only to learn about theories to use in examining their practice but to be able to combine multiple lenses to build up a critical and conceptual versatility. Beyond learning about methods, theories and concepts, sharing perspectives was one factor that contributed to the more expansive views reflected in our results. Participants spoke of the benefits of seeing the approaches taken by their tutors on the postgraduate programme, as well as the descriptions by their international peers of diverse practices and contexts. By engaging in dialogue with teachers from other disciplines, locations and cultures, participants could develop a more grounded and detailed appreciation of the differences between approaches in different settings and what it means to meaningfully contextualise the application of theory to practice. Participants also described the value in having to explicitly articulate their awareness of elements that might otherwise remain implicit (e.g., hierarchies, infrastructure, values, theoretical perspectives).

Teaching, rather than consisting only of instruction, is also part of the wider activity that goes on in classrooms, wards, and other learning environments, in which people develop practices and ways of enacting professional identities (Fenwick et al., 2015; Lave, 1994). Thus, the development of clinicians as teachers involves more than discrete sessions oriented at the technical aspects of teaching (e.g., teacher training), but the creation of space in which to reflect on experiences, to be reflexive about one’s position within complex contexts, and in which to discuss perspectives, methods, theories and their situated application. As part of this, there is likely to be value in those running teacher development programmes such as ours in sharing their own challenges and developmental experiences as teachers, and how we have managed to resolve some of the issues that our students are also grappling with.

Our analysis also helped us to re-examine established conceptions of the relationship between teacher and learning environment. We saw a shift in participants’ perceptions from teachers providing safe or conducive learning environments, demonstrated in the teaching philosophies, towards an appreciation, articulated by some participants in research interviews, of the need to integrate into existing settings and adapt to dynamic contexts (Fawns et al., 2021b). Indeed, where some participants’ early teaching philosophies advocated controlling experiences to support meeting learning objectives, we argue that much important learning in professional education happens the other way around, where the role of teachers is to support learning from unpredictable and uncontrollable experiences, and from diverse settings, contexts and groups of people (as evident during the Covid-19 pandemic). Institutional and cultural constraints play a role in ensuring that the agency of individual educators is checked against collective goals, aspirations, and approaches. In healthcare, practitioners have limited control over their context, and the structures that shape their practice must often be negotiated indirectly (Anya et al., 2018).

### Limitations and reflexivity

Although our participants, by enrolling in a postgraduate programme, may be outliers within the wider population of clinical teachers, they are students, rather than experts, of pedagogy. As such, we cannot say that their conceptions of teaching are representative of expertise. An expanding appreciation of context and the embodiment of educational values is better understood as part of an ongoing development that does not stop once the programme of study is complete. Yet, our students aspire to teach at an advanced level, and it has been interesting for us as educators to reflect on their developing perspectives, many of which mirror our own.

The themes in our results are familiar to us within our experiences and development as teachers and researchers of education. We have surely influenced our participants’ perspectives (indeed, this is our aim as teachers), yet we too learn about education through our own teaching, which includes learning from the diverse settings and descriptions of practice of our students. Our own conceptions of what it means to teach are undoubtedly embedded in, and also shaped by, the programme and this study. Further, as the timeframe of this study extends now over a number of years, it becomes difficult to disentangle what we have learned through the analysis of the data and through our broader experience of teaching clinical educators. Both are part of a greater whole which, while troublesome for the usual processes of research publication, we consider to be healthy for our practice. However, we recognise that other interpretations of our data are possible. As Veen (2021 p.1175) notes, “Abduction has an inherent humility because it acknowledges that, while the conclusion is a sufficient explanation of the observation, it is not necessarily the only possible explanation”. Our aim was to generate an explanation of educators’ developing understandings of teaching, and our thematic framework was developed on the basis of the complexity we saw in the data. It would be beneficial to test and refine the themes generated here with further study aimed at ‘fleshing out’ complexity in relation to the conceptions of developing healthcare educators, for example, by specifically asking about contextual elements, structures, cultures, engagement with materials, etc. It would also be beneficial for others engaged in different teacher development programmes to explore their contexts, producing different interpretations that could serve to compare and contrast with those we have presented here.

It is also problematic to link our participants’ changing understandings to particular methods of postgraduate education. While we, as educators, can shape the learning of our students, it is clear to us that the qualities and experiences that they bring with them are also part of this mix. These parameters feed into the learning community, the dialogue, and the formative and summative activities during the programme. In this sense, the learning within the programme is constituted by the students as much as it is by the teachers. A further note of caution is that these themes do not necessarily represent shifts in our participants’ actual practice. We cannot be sure that any given teacher’s epistemological views are consistent, or translate directly into their teaching practices (see, for example, Olafson & Schraw 2006), and there are many barriers (e.g. time, resources, culture) to teachers being able to enact the kinds of practices they espouse. Indeed, Buehl & Beck (2015) argue that the relationship between teaching beliefs and practices is context-dependent and varies across individuals. However, our participants are well-positioned to shed light on how understandings of teaching change as educators learn more about education. Our focus in this paper has been on the different ways in which ideas about teaching develop, rather than on how that development comes about, although our data do present useful insights about the potential benefits of exposure to multiple theories, methods, and contexts, and constructive dialogue.

## Conclusions

We identified four themes in relation to our participants’ expanding perceptions of what is involved in developing as teachers: repertoire-building; perspective-shifting; embodied practice; and appreciation of context. These themes represent a different way of looking at the development of teacher identity and development, moving away from categorisation of skills and attributes. By building up a repertoire of potential practices and theories, our healthcare educators informed their perspectives on education. The adoption of new and multiple perspectives could help clinical teachers see past particular methods or conceptions and develop embodied approaches to attuning their practice to particular situations. Finally, some participants indicated an increasing awareness of the subtleties of cultural, political and social factors in which teaching practice is embedded. This theme recognises the considerable constraints within which teaching practice can be situated, and the impossibility of direct control over many important aspects of their settings, as well as the kinds of work necessary to bring about change in complex systems. The final two themes, in particular, the ongoing development of teachers that does not stop once any programme of study is complete. Postgraduate study can support these developments by creating space in which to try things out, reflect, and discuss with a diverse community of peers.
