# Correction: Making space to learn about teaching: expanding teaching horizons through postgraduate education

**DOI:** 10.1007/s10459-022-10199-3

**Published:** 2022-12-29

**Authors:** Gillian Aitken, Tim Fawns, Katey Warran, Derek Jones

**Affiliations:** Edinburgh Medical School: Medical Education, University of Edinburgh, Chancellor’s Building, EH16 4SB Edinburgh, Scotland

**Correction to:** **Advances in Health Sciences Education**

10.1007/s10459-022-10144-4

In the above mentioned publication, the penultimate author’s surname contained a typo as it should have read Warran.

The original article has been corrected.

